# Transcriptional profiling of PPARα−/− and CREB3L3−/− livers reveals disparate regulation of hepatoproliferative and metabolic functions of PPARα

**DOI:** 10.1186/s12864-019-5563-y

**Published:** 2019-03-11

**Authors:** Philip M. M. Ruppert, Jong-Gil Park, Xu Xu, Kyu Yeon Hur, Ann-Hwee Lee, Sander Kersten

**Affiliations:** 1 Nutrition, Metabolism and Genomics group, Division of Human Nutrition and Health, Wageningen University, Stippeneng 4, 6708WE, Wageningen, the Netherlands; 2 Department of Pathology and Laboratory Medicine, Weill Cornell Medicine, 1300 York Ave, New York, NY 10065 USA; 3 Department of Medicine, Samsung Medical Center, Sungkyunkwan University School of Medicin, Seoul, South Korea; 4 Present address: Biotherapeutics Translational Research Center, Korea Research Institute of Bioscience and Biotechnology, Daejeon, 34141 South Korea; 5 Present address: Division of Gastroenterology and Hepatology, Joan & Sanford I. Weill Department of Medicine, Weill Cornell Medicine, New York, NY 10021 USA; 6 Present address: Regeneron Pharmaceuticals, 777 Old Saw Mill River Rd, Tarrytown, NY 10591 USA

**Keywords:** Liver, CREB3L3, PPARα, Fasting, Ketogenic diet, Transcriptomics

## Abstract

**Background:**

Peroxisome Proliferator-Activated receptor α (PPARα) and cAMP-Responsive Element Binding Protein 3-Like 3 (CREB3L3) are transcription factors involved in the regulation of lipid metabolism in the liver. The aim of the present study was to characterize the interrelationship between PPARα and CREB3L3 in regulating hepatic gene expression. Male wild-type, PPARα−/−, CREB3L3−/− and combined PPARα/CREB3L3−/− mice were subjected to a 16-h fast or 4 days of ketogenic diet. Whole genome expression analysis was performed on liver samples.

**Results:**

Under conditions of overnight fasting, the effects of PPARα ablation and CREB3L3 ablation on plasma triglyceride, plasma β-hydroxybutyrate, and hepatic gene expression were largely disparate, and showed only limited interdependence. Gene and pathway analysis underscored the importance of CREB3L3 in regulating (apo)lipoprotein metabolism, and of PPARα as master regulator of intracellular lipid metabolism. A small number of genes, including *Fgf21* and *Mfsd2a*, were under dual control of PPARα and CREB3L3. By contrast, a strong interaction between PPARα and CREB3L3 ablation was observed during ketogenic diet feeding. Specifically, the pronounced effects of CREB3L3 ablation on liver damage and hepatic gene expression during ketogenic diet were almost completely abolished by the simultaneous ablation of PPARα. Loss of CREB3L3 influenced PPARα signalling in two major ways. Firstly, it reduced expression of PPARα and its target genes involved in fatty acid oxidation and ketogenesis. In stark contrast, the hepatoproliferative function of PPARα was markedly activated by loss of CREB3L3.

**Conclusions:**

These data indicate that CREB3L3 ablation uncouples the hepatoproliferative and lipid metabolic effects of PPARα. Overall, except for the shared regulation of a very limited number of genes, the roles of PPARα and CREB3L3 in hepatic lipid metabolism are clearly distinct and are highly dependent on dietary status.

**Electronic supplementary material:**

The online version of this article (10.1186/s12864-019-5563-y) contains supplementary material, which is available to authorized users.

## Background

The liver plays a critical role in the metabolic response to changes in the diet. An important regulatory mechanism in the control of metabolism is via changes in the expression of relevant genes. Indeed, changes in nutrient composition and nutrient availability trigger profound changes in the hepatic expression of numerous genes involved in glucose and lipid metabolism. An important transcription factor that is involved in the adaptive response to changes in nutrient supply is PPARα [1, 2]. PPARα is a member of the family of nuclear receptors and part of the subfamily of Peroxisome Proliferators Activated Receptors, which also includes PPARβ/δ and PPARγ [3]. The PPARs share a common mode of action that involves heterodimerization with the nuclear receptor RXR (Retinoid X Receptor), followed by binding of the PPAR-RXR complex to specific DNA sequences in the regulatory regions of target genes [4–6]. Activation of transcription is triggered by binding of a ligand, which include fatty acids and fatty acid derivatives such as eicosanoids and oxidized fatty acids, as well as a variety of synthetic compounds collectively referred to as peroxisome proliferators [7].

Evidence abounds indicating that PPARα is crucial for the transcriptional regulation of hepatic lipid metabolism during fasting. Indeed, studies employing expression profiling of whole body or liver-specific PPARα−/− mice have demonstrated that PPARα induces the expression of hundreds of genes involved in nearly every branch of hepatic lipid metabolism [8–12]. Hence, PPARα can be aptly described as the master regulator of hepatic lipid metabolism, especially under conditions of elevated hepatic lipid load, as occurs during fasting, high fat feeding, and a ketogenic diet. In line with this notion, the absence of PPARα during fasting leads to a host of metabolic disturbances, including a fatty liver, elevated plasma non-esterified fatty acids, hypoglycemia and hypoketonemia [8–12].

Cyclic AMP-responsive element-binding protein 3 Like 3 (CREB3L3, encoded by *Creb3l3*) is a bZiP transcription factor that is highly expressed in the liver [13]. CREB3L3 is produced as an ER precursor form and is proteolytically activated in the Golgi to liberate the N-terminal portion that functions as a transcriptional activator [13]. Growing evidence implicates CREB3L3 in the regulation of glucose and lipid metabolism in the liver [14]. Specifically, CREB3L3 has been shown to stimulate gluconeogenesis [15] and glycogenolysis [16], plasma triglyceride clearance [17], and lipid droplet formation [18].

Both PPARα and CREB3L3 are activated in the liver by fasting and play important roles in the utilization of fatty acids for energy in the fasted state [8–10, 19]. Several lines of evidence point to an interaction between PPARα- and CREB3L3-mediated gene regulation. First, PPARα has been shown to regulate CREB3L3 expression in human and mouse hepatocytes [20], likely via a PPRE located upstream of exon 3 [21], indicating that *Creb3l3* is a direct PPARα target gene. Second, there is strong evidence that PPARα and CREB3L3 cooperate in the regulation of certain genes such as *Fgf21*, encoding Fibroblast Growth Factor 21*.* Specifically, PPARα and CREB3L3 physically interact to form a complex that binds to an integrated CRE-PPAR-responsive element-binding motif in the *Fgf21* gene promoter [22]. The physical interaction between PPARα and CREB3L3 is enhanced by fasting and dependent on CREB3L3 acetylation at K294 [23]. More recently, it was shown that during fasting, PPARα and CREB3L3 also cooperate in the stimulation of hepatic gluconeogenesis by targeting genes such as *Pck1*, encoding Phosphoenolpyruvate Carboxykinase 1 [16]. Other genes that are under dual control of PPARα and CREB3L3 in liver include *Cidec*, encoding Cell Death Inducing DFFA Like Effector C [18, 24]. The data presented above suggest that part of the effects of PPARα may be mediated by CREB3L3 and point towards cooperativity in gene regulation by PPARα and CREB3L3. Based on the analysis of the phenotype of single and combined PPARα−/− and CREB3L3−/− mice, it was proposed that CREB3L3 co-operates with PPARα by directly and indirectly regulating the expression of genes involved in fatty acid oxidation and ketogenesis [25].

To further characterize the cooperativity between PPARα and CREB3L3 in hepatic gene regulation, we studied the effect of PPARα and CREB3L3 ablation, either individually or combined, on overall hepatic gene regulation using whole genome expression profiling, in mice after a 16-h fast and after 4 days of ketogenic diet.

## Results

### Effect of PPARα and/or CREB3L3 ablation on fasting plasma metabolites

**Fig. 1:**
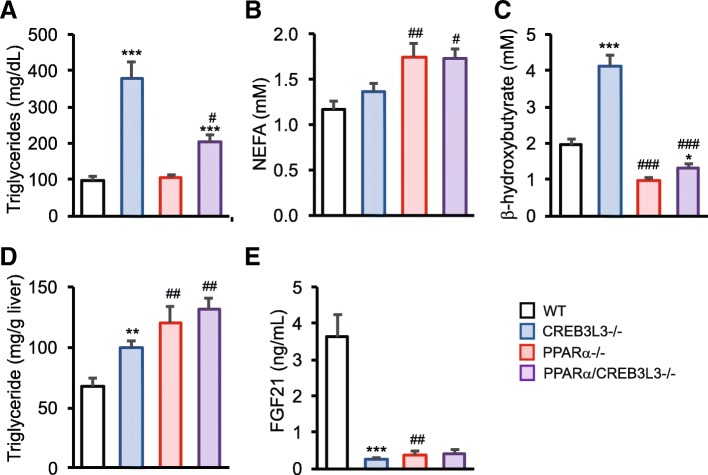
Effect of single and combined PPARα and CREB3L3 deficiency on metabolic parameters. PPARα−/−, CREB3L3−/− and combined PPARα/CREB3L3−/− mice were subjected to a 16-h fast. **a** Plasma triglycerides. WT, *n* = 9; CREB3L3−/−, *n* = 11; PPARα−/−, *n* = 7; PPARα/CREB3L3−/−, *n* = 6. **b** Plasma non-esterified fatty acids (NEFA). WT, *n* = 9; CREB3L3−/−, *n* = 11; PPARα−/−, *n* = 8; PPARα/CREB3L3−/−, *n* = 6. **c** Plasma β-hydroxybutyrate. WT, *n* = 9; CREB3L3−/−, *n* = 11; PPARα−/−, *n* = 8; PPARα/CREB3L3−/−, *n* = 6. **d** Hepatic triglycerides. WT, *n* = 7; CREB3L3−/−, *n* = 9; PPARα−/−, n = 6; PPARα/CREB3L3−/−, *n* = 6. **e** Plasma Fibroblast Growth Factor 21. WT, *n* = 5; CREB3L3−/−, *n* = 8; PPARα−/−, *n* = 4; PPARα/CREB3L3−/−, *n* = 4. Error bars represent SEM. Asterisk indicates significant effect of CREB3L3 deficiency in wild-type mice (blue vs. white bar) and in PPARα mice (purple vs red bar) according to Student’s t-test (*P < 0.05, **P < 0.01, ***P < 0.001). Pound sign indicates significant effect of PPARα deficiency in wild-type mice (red vs. white bar) and in CREB3L3 mice (purple vs. blue bar) according to Student’s t-test (#*P* < 0.05, ##*P* < 0.01, ###*P* < 0.001)

To study the potential interaction between PPARα and CREB3L3 in metabolic regulation in the fasted state, we first performed basic metabolic measurements in wild-type, PPARα−/−, CREB3L3−/−, and combined PPARα/CREB3L3−/− mice after 16 h of fasting. Plasma triglyceride levels were markedly elevated in the CREB3L3−/−, but not in the PPARα−/− mice (Fig. 1a). The hypertriglyceridemia in CREB3L3−/− mice was improved by the simultaneous ablation of PPARα, suggesting functional antagonism between PPARα and CREB3L3 in plasma triglyceride regulation (Fig. 1a). As previously shown [9], PPARα ablation significantly increased plasma non-esterified fatty acid (NEFA) levels (Fig. 1b), and decreased β-hydroxybutyrate levels (Fig. 1c). In agreement with our previous report [19], NEFA and β-hydroxybutyrate levels were elevated in CREB3L3−/− mice, while levels in PPARα/CREB3L3−/− mice were similar to those in PPARα−/− mice (Fig. 1b and c), suggesting a dominant effect of PPARα ablation. Interestingly, liver triglyceride levels were elevated in both PPARα−/− and CREB3L3−/− mice compared with wild-type mice and were highest in the combined PPARα/CREB3L3−/− mice (Fig. 1d). Plasma FGF21 levels were dramatically lower in PPARα−/−, CREB3L3−/−, and PPARα/CREB3L3−/− mice as compared with wild-type mice (Fig. 1e). These data indicate the pronounced impact of PPARα and CREB3L3 deficiency on metabolic regulation during fasting.

### Effects of PPARα and CREB3L3 ablation on hepatic gene expression in the fasted state are largely independent

**Fig. 2:**
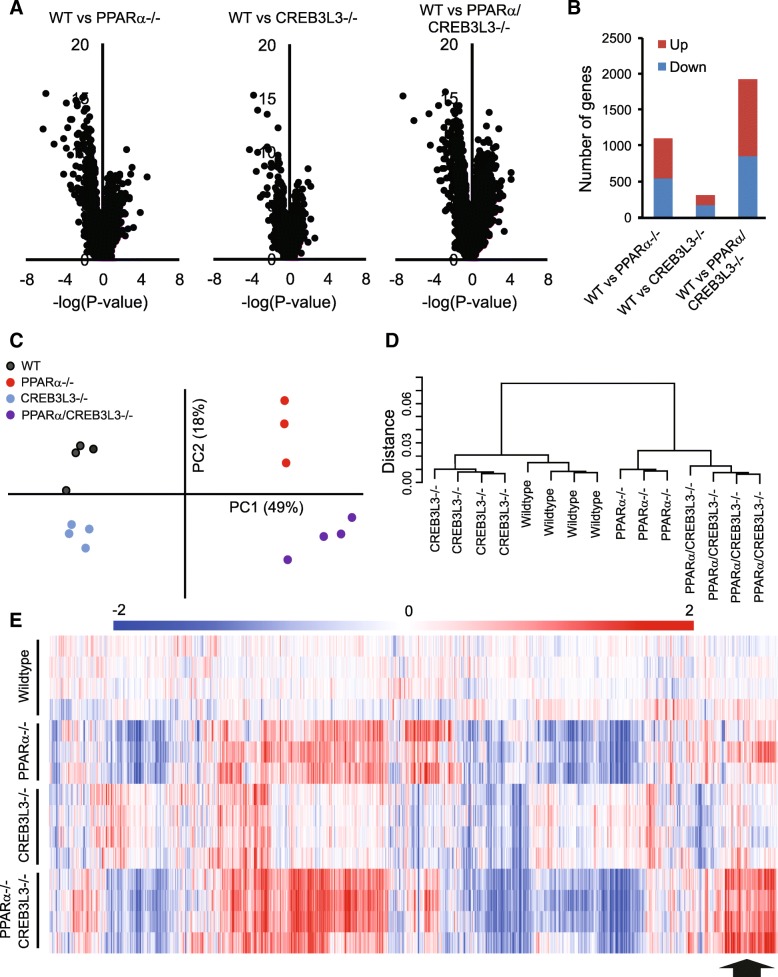
Largely independent effect of PPARα and CREB3L3 deficiency on hepatic gene expression in the fasted state. **a** Volcano plot showing the relation between mean signal log ratio (^2^log[fold-change], x-axis) and the -^10^log of the IBMT *P*-value (y-axis) for the comparison between wild-type mice and PPARα−/− mice, CREB3L3−/− mice and combined PPARα/CREB3L3−/− mice after a 16-h fast. **b** Number of genes meeting significance criteria (fold change<− 1.2 or > 1.2 and IBMT *P* < 0.001) for the comparison between wild-type mice and PPARα−/− mice, CREB3L3−/− mice and combined PPARα/CREB3L3−/− mice after a 16-h fast. Principle component analysis (**c**) and hierarchical clustering (**d**) of transcriptomics data from livers of wild-type, PPARα−/−, CREB3L3−/−, and combined PPARα/CREB3L3−/− mice after a 16-h fast. Distance criteria are based on Pearson correlation with average linkage. An IQR (Inter Quartile Range) filter of 0.5 was applied. **e** Hierarchical biclustering of samples and genes visualized in a heatmap. Data were centered on wild-type mean. Clustering was done based on Pearson correlation with average linkage. Red indicates upregulated, blue indicates downregulated. The region in the heatmap that is suggestive of synergistic regulation by PPARα and CREB3L3 deficiency is indicated by a grey arrow

To study the potential interaction between PPARα and CREB3L3 in hepatic gene regulation in the fasted state, whole genome expression analysis was performed on liver samples of the four groups of mice after 16 h of fasting. To study the magnitude of the effect of PPARα and CREB3L3 ablation on liver gene expression, we performed Volcano plot analysis. Interestingly, the effects of PPARα ablation were much more pronounced as compared to CREB3L3 ablation (Fig. 2a). The combined ablation of PPARα and CREB3L3 had the most significant effect on gene regulation, pointing to a potential additive or synergistic effect of PPARα and CREB3L3 ablation on hepatic gene expression. Analysis of the number of significantly changed genes showed that in the fasted state, loss of PPARα altered the expression of 1097 genes, of which 553 genes were upregulated and 544 genes were downregulated (Fig. 2b). Loss of CREB3L3 altered expression of 312 genes, of which 134 genes were upregulated and 178 genes were downregulated. Combined loss of PPARα and CREB3L3 altered the expression of 1917 genes, of which 1064 genes were upregulated and 853 genes were downregulated (Fig. 2b). The fact that the number of significantly changed genes in the combined PPARα/CREB3L3−/− mice exceeds the sum of significantly changed genes in the PPARα−/− and CREB3L3−/− mice suggests a modest synergistic effect of PPARα and CREB3L3 deficiency on hepatic gene regulation, dominated by PPARα.

To study the potential similarity between the effect of PPARα and CREB3L3 deficiency on liver gene expression, we performed principle component analysis (Fig. 2c) and hierarchical clustering (Fig. 2d). Both analyses indicated the separate clustering of the four experimental groups, with limited variation between the individual mice in each group, and the more pronounced effect of PPARα deficiency compared to CREB3L3 deficiency. In addition, both analyses showed that the whole genome effects of combined PPARα/CREB3L3 deficiency are largely taken up by PPARα deficiency (Fig. 2c, d).

Hierarchical biclustering of samples and genes visualized in a heatmap further supported the conclusions reached above, showing the much more pronounced effect of PPARα deficiency on hepatic gene expression and the more significant contribution of PPARα towards the effect of combined PPARα/CREB3L3 deficiency (Fig. 2e). The heat map also shows that for certain genes, the effects of PPARα and CREB3L3 deficiency are additive and seemingly independent, whereas for other genes the effect of PPARα and CREB3L3 deficiency appears to be synergistic and thus dependent.

### A limited number of genes is commonly downregulated by PPARα and CREB3L3 deficiency in liver during fasting

**Fig. 3:**
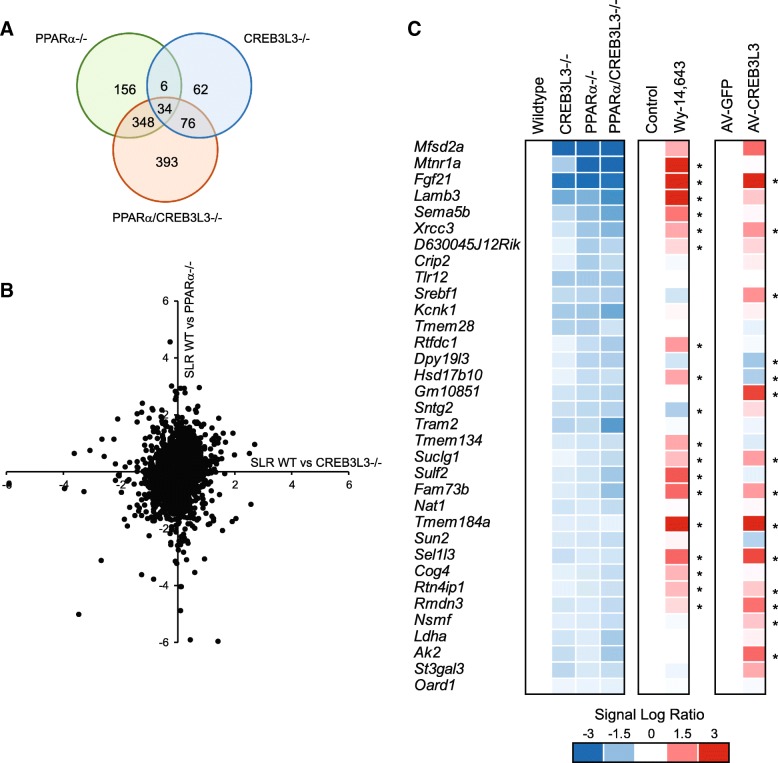
Limited overlap in effects of PPARα and CREB3L3 deficiency on hepatic gene expression in the fasted state. **a** Venn diagram showing overlap in downregulated genes in PPARα−/−, CREB3L3−/−, and combined PPARα/CREB3L3−/− mice, in comparison with wild-type mice (IBMT P-value< 0.001). **b** Correlation plot showing gene expression changes in CREB3L3−/− mice in relation to wild-type mice (x-axis) and in PPARα−/− mice in relation to wild-type mice (y-axis) (expressed as signal log ratio, SLR). **c** Comparative gene expression analysis in liver of wild-type, PPARα−/−, CREB3L3−/−, and combined PPARα/CREB3L3−/− mice after 16-h fast. For comparison, gene expression changes in mouse liver after PPARα activation by 5-day treatment with the agonist Wy-14,643 are shown (GSE8316) [51], as well as gene expression changes in mouse liver after adenoviral-mediated over expression of CREB3L3. Asterisk indicates significantly different from control conditions according to IBMT *P*-value< 0.001. The 34 genes shown are the commonly downregulated genes in livers of PPARα−/−, CREB3L3−/−, and PPARα/CREB3L3−/− mice in the fasted state (IBMT *P*-value< 0.001)

The Venn diagram of significantly downregulated genes (Fig. 3a) and scatter plot analysis (Fig. 3b) confirmed that, in general, the effects of PPARα and CREB3L3 deficiency are disparate, with only a limited number of genes showing similar regulation in PPARα−/− and CREB3L3−/− mice. The list of 34 genes that were significantly downregulated in livers of PPARα−/−, CREB3L3−/−, and PPARα/CREB3L3−/− mice is presented in Additional file 1: Table S1. This list includes *Fgf21*, which is known to be under dual control of PPARα and CREB3L3, and *Mfsd2a*, a gene involved in lysophospholipid transport that is known to be under control of PPARα but not CREB3L3 [26, 27]. To examine whether any of these 34 genes may be directly regulated by PPARα, we determined the effect of the PPARα agonist Wy-14,643 on gene expression in the liver (Fig. 3c). To examine whether any of these 34 genes may be directly regulated by CREB3L3, we determined the effect of adenoviral-mediated CREB3L3 overexpression on gene expression in the liver (Fig. 3c). The results suggest that several of the 34 genes may be direct targets of both PPARα and CREB3L3, as they are markedly upregulated by PPARα and CREB3L3 activation. These genes include *Fgf21*, *Mfsd2a, Xrcc3*, *Suclg1*, *Tmem184a* and *Sel1l3*.

**Fig. 4:**
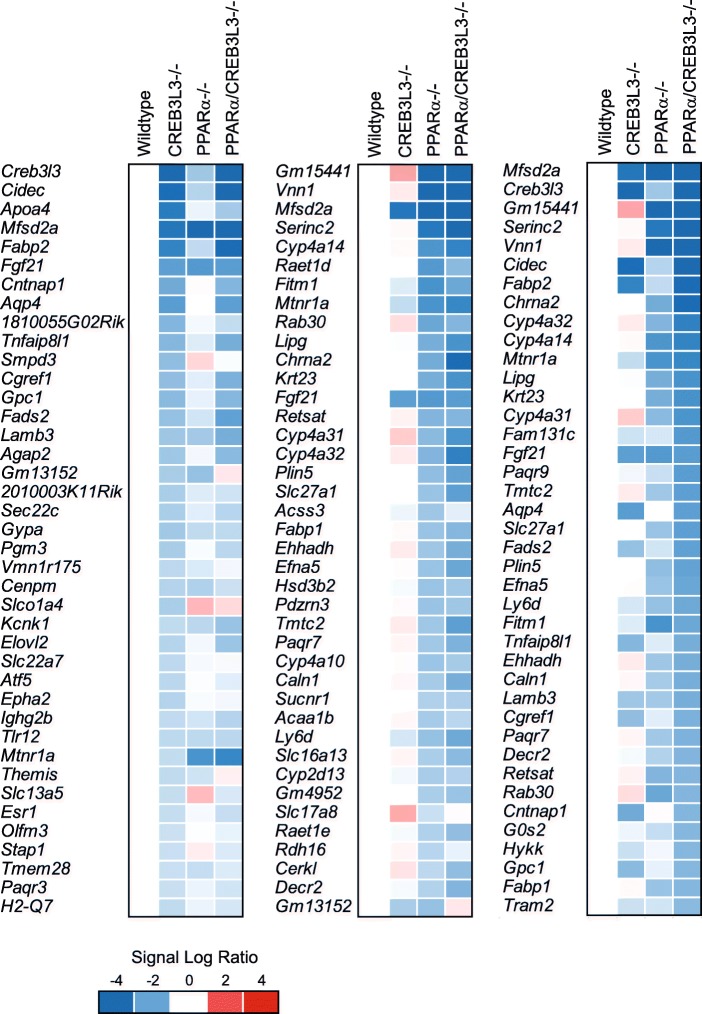
PPARα and CREB3L3 mostly regulate distinct genes in liver in the fasted state. Comparative gene expression analysis in liver of wild-type, PPARα−/−, CREB3L3−/−, and combined PPARα/CREB3L3−/− mice after a 16-h fast, showing the top 50 most highly downregulated genes in CREB3L3−/− mice (left panel), PPARα−/− mice (middle panel), and combined PPARα/CREB3L3−/− (right panel) in the form a heatmap, using the mean expression of each group. Red indicates upregulated, blue indicates downregulated

To further explore the differential impact of PPARα and CREB3L3 deficiency on hepatic gene expression, the top 40 most significantly downregulated genes in each condition (PPARα−/−, CREB3L3−/−, and PPARα/CREB3L3−/−) were taken and visualized in a heatmap (Fig. 4). The top 40 list of most significantly downregulated genes in the CREB3L3−/− mice contains the known CREB3L3 targets *Cidec*, *Apoa4*, and *Fgf21*. A relatively large portion of the genes downregulated in the CREB3L3−/− mice were also downregulated in the PPARα−/− mice and, especially, in the combined PPARα/CREB3L3−/− mice. For PPARα, only a very small portion of the genes downregulated in the PPARα−/− mice were also downregulated in the CREB3L3−/− mice, the exception being *Fgf21*, *Mfsd2a*, and *Mtnr1a*. Other typical PPARα target genes such as *Retsat*, *Cy4a14*, *Plin5*, *Fabp1*, *Acaa1b* and *Ehhadh* were exclusively downregulated in the PPARα−/− mice. For nearly all genes shown, the downregulation in the PPARα−/− mice was copied in the combined PPARα/CREB3L3−/− mice. The top 40 list of most significantly downregulated genes in the PPARα/CREB3L3−/− mice represents a combination of genes mainly controlled by PPARα (*Vnn1*, *Cyp4a14*, *Krt23*, *Slc27a1*), by CREB3L3 (*Cidec*, *Fabp2*), or both (*Fgf21*, *Mfsd2a*, *Mtnr1a*) (Fig. 4).

**Fig. 5:**
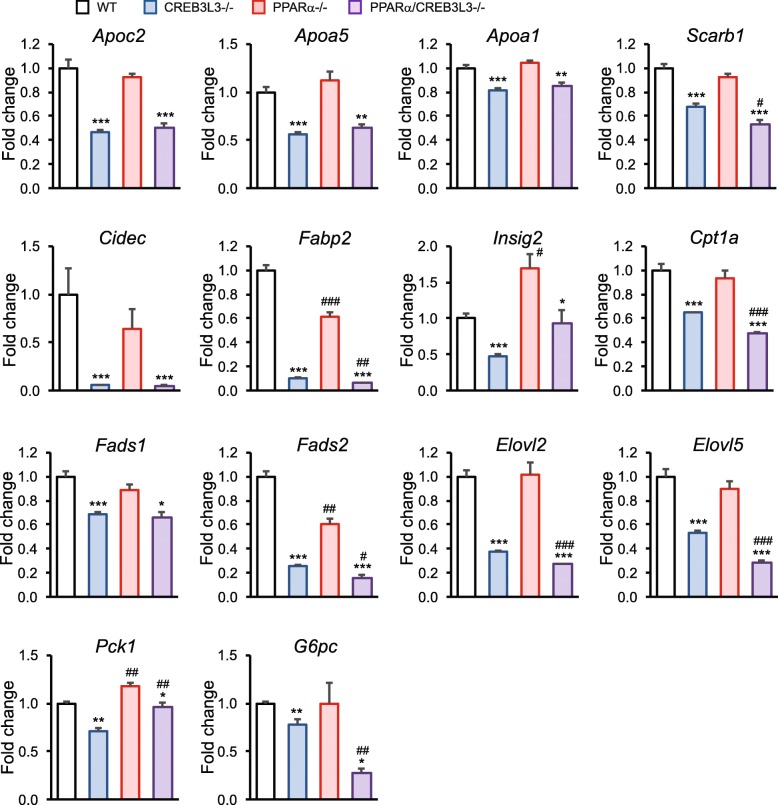
Microarray gene expression of selected genes previously shown to be under control of CREB3L3. All are significantly downregulated in liver of fasted CREB3L3−/− mice as compared to fasted wild-type mice (IBMT *P*-value< 0.001). Asterisk indicates significant effect of CREB3L3 deficiency in wild-type mice (blue vs. white bar) and in PPARα mice (purple vs red bar) according to Student’s t-test (*P < 0.05, **P < 0.01, ***P < 0.001). Pound sign indicates significant effect of PPARα deficiency in wild-type mice (red vs. white bar) and in CREB3L3 mice (purple vs. blue bar) according to Student’s t-test (#*P* < 0.05, ##*P* < 0.01, ###*P* < 0.001)

A limited number of genes has been identified as direct or putative target of CREB3L3. The expression levels of these genes are illustrated in Fig. 5, showing that CREB3L3 deficiency leads to significant downregulation of genes involved in lipoprotein metabolism (*Apoc2, Apoa5, Apoa1, Scarb1*), lipid storage (*Cidec*), fatty acid binding (*Fabp2*), fatty acid desaturation and elongation (*Fads1, Fads2, Elovl2, Elovl5*), gluconeogenesis (*Pck1, G6pc*), and fatty acid oxidation (*Cpt1a*). Most of these genes were not or minimally affected by PPARα deficiency. Together, these data indicate that in the fasted state CREB3L3 and PPARα regulate different sets of genes, with some notable exceptions, suggesting that the transcription factors largely operate independently.

### CREB3L3 deficiency leads to downregulation of genesets related to lipoprotein and lipid transport

**Fig. 6:**
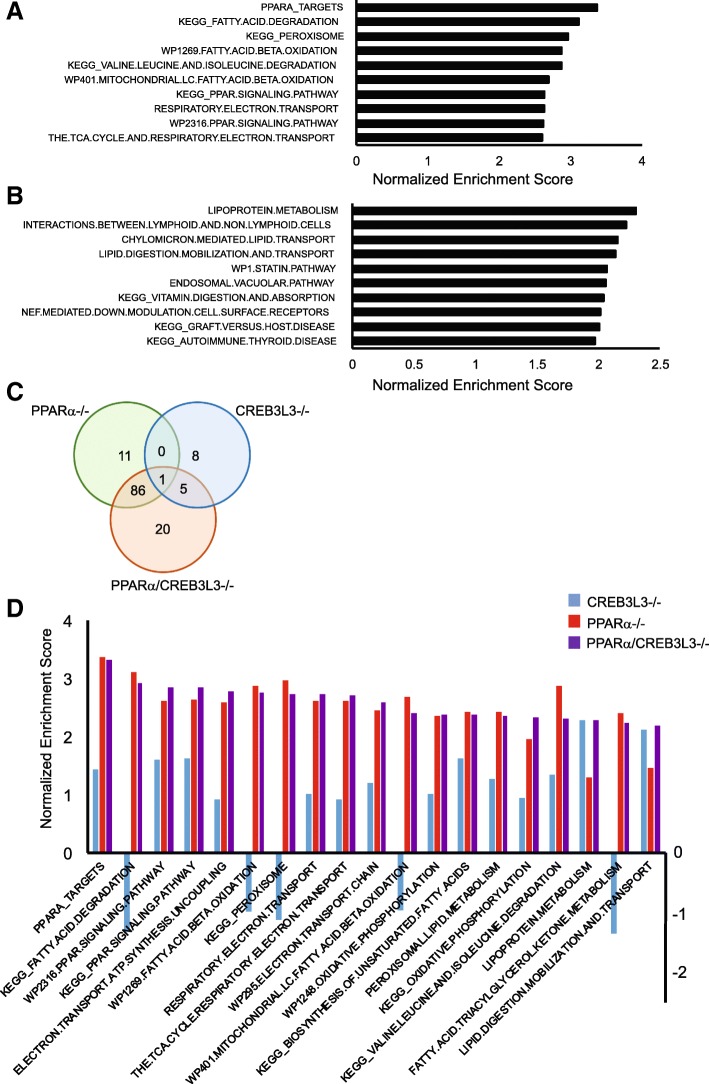
PPARα and CREB3L3 regulate distinct pathways in the liver in the fasted state. **a** Top 10 downregulated genesets in liver of PPARα−/− compared with wild-type mice, determined by gene set enrichment analysis. **b** Top 10 downregulated genesets in liver of CREB3L3−/− compared with wild-type mice, determined by gene set enrichment analysis. Genesets were ranked according to normalized enrichment score (NES). **c** Venn diagram showing overlap in downregulated genesets (FDR q-value< 0.1) in PPARα−/−, CREB3L3−/−, and combined PPARα/CREB3L3−/− mice, in comparison with wild-type mice. **d** Top 20 downregulated gene sets in liver of combined PPARα/CREB3L3−/− mice compared with wild-type mice, determined by gene set enrichment analysis and ranked according to NES (purple). The NES of the same genesets for the comparison between wild-type and PPARα−/− (red) or CREB3L3−/− (blue) mice is shown as well

To gain more insight into the functional differences between PPARα and CREB3L3 deficiency, we compared the effects of PPARα and CREB3L3 deficiency at the level of pathways using geneset enrichment analysis (Fig. 6a). Deficiency of PPARα led to the downregulation of numerous genesets that are known to be controlled by PPARα, mainly representing genesets related to peroxisomal and mitochondrial fatty acid catabolism and the electron transport chain. By contrast, deficiency of CREB3L3 led to the downregulation of genesets related to lipoprotein and lipid transport, as well as several genesets connected to immunity (Fig. 6b). At the pathway level, minimal overlap was observed between the effect of PPARα and CREB3L3 deficiency (Fig. 6c). In fact, out of 98 genesets that were significantly downregulated in PPARα−/− mice, only one geneset, named branched chain amino acid catabolism, was also downregulated in the CREB3L3−/− mice (Fig. 6c). The commonly enriched genes within the geneset branched chain amino acid catabolism included *Auh*, *Hibch*, *Hibadh*, *Acad8*, *Ivd*, and *Hsd17B10*.

Consistent with the notion that the effects of combined PPARα/CREB3L3 deficiency are largely taken up by PPARα deficiency, the far majority of genesets downregulated in the combined PPARα/CREB3L3−/− mice were also downregulated in the PPARα−/− mice (Fig. 6d). Indeed, the enrichment scores of the most highly downregulated genesets in the combined PPARα/CREB3L3−/− mice were very similar in the single PPARα−/− mice, suggesting that the functional impact of combined PPARα/CREB3L3−/− deficiency is mostly accounted for by deficiency of PPARα. The exception were two genesets related to lipoprotein and lipid transport, which had similar enrichment scores in the combined PPARα/CREB3L3−/− mice and single CREB3L3−/− mice (Fig. 6d), suggesting that the regulation of these two genesets is driven by CREB3L3 deficiency.

Deficiency of PPARα led to the upregulation of genesets related to the unfolded protein response and inflammatory signalling (Additional file 1: Figure S1A). By contrast, deficiency of CREB3L3 led to upregulation of genesets related to cholesterol synthesis and protein translation (Additional file 1: Figure S1B). Consistent with this result, genes involved in cholesterol metabolism feature prominently among the top 40 most highly upregulated genes in CREB3L3−/− mice (Additional file 1: Figure S1C).

Overall, the above analyses indicate that the effects of PPARα and CREB3L3 deficiency on hepatic gene expression during fasting are very distinct. Only a limited number of genes is under regulation of both PPARα and CREB3L3. The PPARα/CREB3L3−/− mice reflect the combined effect of especially PPARα and to a lesser extent CREB3L3 deficiency, showing a minor degree of synergism.

### Effect of PPARα and/or CREB3L3 deficiency on plasma metabolites during ketogenic diet

**Fig. 7:**
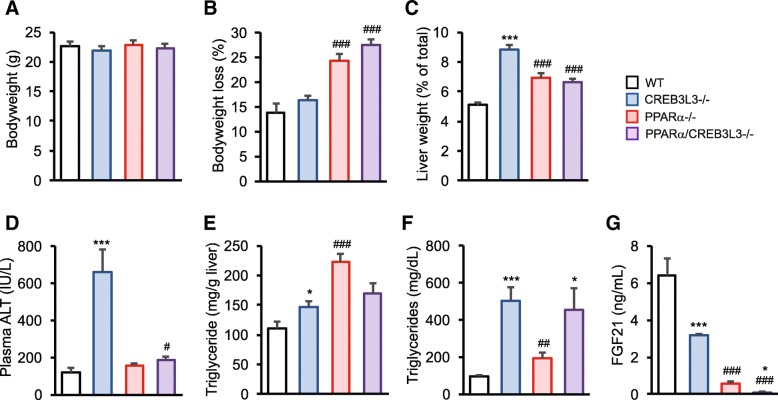
Effect of single and combined PPARα and CREB3L3 deficiency on metabolic parameters. PPARα−/−, CREB3L3−/− and combined PPARα/CREB3L3−/− mice were subjected to a 4 day ketogenic diet. **a** Bodyweight before the ketogenic diet. WT, *n* = 15; CREB3L3−/−, *n* = 12; PPARα−/−, *n* = 9; PPARα/CREB3L3−/−, *n* = 10. **b** Percentage bodyweight loss caused by the ketogenic diet. WT, n = 15; CREB3L3−/−, n = 12; PPARα−/−, *n* = 10; PPARα/CREB3L3−/−, *n* = 10. **c** Liver weight as percentage of total bodyweight. WT, n = 15; CREB3L3−/−, n = 12; PPARα−/−, *n* = 9; PPARα/CREB3L3−/−, *n* = 10. **d** Plasma alanine aminotransferase activity. WT, n = 15; CREB3L3−/−, *n* = 12; PPARα−/−, *n* = 5; PPARα/CREB3L3−/−, *n* = 6. **e** Hepatic triglycerides. WT, *n* = 10; CREB3L3−/−, *n* = 7; PPARα−/−, *n* = 5; PPARα/CREB3L3−/−, *n* = 6. **f** Plasma triglycerides. WT, *n* = 10; CREB3L3−/−, *n* = 7; PPARα−/−, *n* = 5; PPARα/CREB3L3−/−, *n* = 5. **g** Plasma Fibroblast Growth Factor 21. WT, n = 9; CREB3L3−/−, *n* = 7; PPARα−/−, *n* = 9; PPARα/CREB3L3−/−, n = 5. Error bars represent SEM. Asterisk indicates significant effect of CREB3L3 deficiency in wild-type mice (blue vs. white bar) and in PPARα mice (purple vs red bar) according to Student’s t-test (*P < 0.05, **P < 0.01, ***P < 0.001). Pound sign indicates significant effect of PPARα deficiency in wild-type mice (red vs. white bar) and in CREB3L3 mice (purple vs. blue bar) according to Student’s t-test (#*P* < 0.05, ##*P* < 0.01, ###*P* < 0.001)

To further explore the cooperativity between PPARα and CREB3L3 in hepatic gene regulation, we compared the effect of PPARα and CREB3L3 deficiency under the condition of a ketogenic diet. Previously, this diet was shown to provoke a pronounced hepatic phenotype in CREB3L3−/− mice, characterized by hepatomegaly and signs of steatohepatitis [19, 25]. No difference in bodyweight between the four genotypes was observed before the start of the study (Fig. 7a). Four days of ketogenic diet induced pronounced weight loss in all groups, which was most pronounced in the PPARα−/− mice and combined PPARα/CREB3L3−/− mice (Fig. 7b). Interestingly, compared to the wild-type mice, the liver to body weight ratio was modestly increased in the PPARα−/− mice and combined PPARα/CREB3L3−/− mice, yet was highest in the CREB3L3−/− mice, suggesting hepatomegaly (Fig. 7c) [19, 25]. Compared to the other three groups, CREB3L3−/− mice fed a ketogenic diet for 4 days also exhibited markedly elevated plasma alanine aminotransferase (ALT) activity (Fig. 7d), suggesting liver damage. Plasma ALT levels were below 30 IU/L in all groups before starting the ketogenic diet (not shown). Elevated plasma ALT was accompanied by elevated liver and plasma triglycerides in CREB3L3−/− mice (Fig. 7e,f). These parameters were also increased in the PPARα−/− mice. Plasma FGF21 levels followed a very different pattern and were about 50% decreased in the CREB3L3−/− mice, more than 90% decreased in the PPARα−/− mice, and nearly 99% decreased in the PPARα/CREB3L3−/− mice (Fig. 7g). Overall, these data are in line with a previous report [25].

**Fig. 8:**
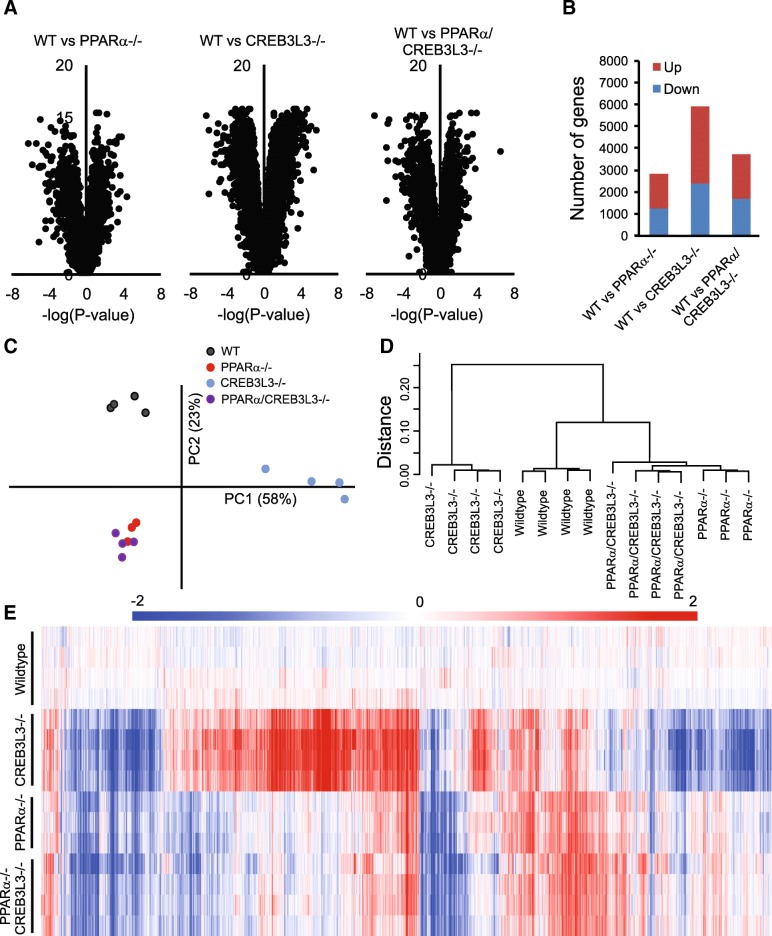
PPARα deficiency mitigates effect of CREB3L3 deficiency on hepatic gene expression during ketogenic diet. **a** Volcano plot showing the relation between signal log ratio (^2^log[fold-change], x-axis) and the -^10^log of the IBMT *P*-value (y-axis) for the comparison between wild-type mice and PPARα−/− mice, CREB3L3−/− mice and combined PPARα/CREB3L3−/− mice after 4 days of ketogenic diet. **b** Number of genes meeting significance criteria (fold change<− 1.2 or > 1.2 and IBMT *P* < 0.001) for the comparison between wild-type mice and PPARα−/− mice, CREB3L3−/− mice and combined PPARα/CREB3L3−/− mice after 4 days of ketogenic diet. Principle component analysis (**c**) and hierarchical clustering (**d**) of transcriptomics data from liver of wild-type, PPARα−/−, CREB3L3−/−, and combined PPARα/CREB3L3−/− mice after a 4 day ketogenic diet. **e** Hierarchical biclustering of samples and genes visualized in a heatmap. An IQR (Inter Quartile Range) filter of 0.5 was applied. Red indicates upregulated, blue indicates downregulated

To study the magnitude of the effect of PPARα and CREB3L3 deficiency during ketogenic diet on liver gene expression, we performed Volcano plot analysis (Fig. 8a). In contrast to what was observed in the fasted state, the effects of CREB3L3 deficiency during ketogenic diet were more pronounced as compared to PPARα deficiency. Strikingly, the effect of combined deficiency of PPARα and CREB3L3 on hepatic gene expression was less pronounced as compared to deficiency of only CREB3L3. Analysis of the number of significantly changed genes showed that loss of CREB3L3 altered the expression of 5878 genes, of which 3490 genes were upregulated and 2388 genes were downregulated (Fig. 8b). Loss of PPARα altered expression of 2843 genes, of which 1616 genes were upregulated and 1227 genes were downregulated. Combined loss of PPARα and CREB3L3 altered the expression of 3707 genes, of which 1996 genes were upregulated and 1711 genes were downregulated. These observations indicate that deficiency of PPARα mitigates the effect of CREB3L3 deficiency on hepatic gene expression.

### Effects of CREB3L3 deficiency on hepatic gene expression during ketogenic diet are dependent on PPARα

To study the similarity between the three different genetic models in liver gene expression, we performed principle component analysis (Fig. 8c) and hierarchical clustering (Fig. 8d). Principle component analysis and hierarchical clustering of samples showed that the CREB3L3−/− mice formed a distinct cluster, underscoring the profound effect of CREB3L3 deficiency on hepatic gene expression during ketogenic diet. Surprisingly, the PPARα−/− mice and combined PPARα/CREB3L3−/− mice clustered together and were very distinct from the CREB3L3−/− mice. Hierarchical biclustering of samples and genes visualized in a heatmap further confirmed that at the level of hepatic gene expression, the PPARα−/− mice and combined PPARα/CREB3L3−/− mice were nearly indistinguishable, whereas the CREB3L3−/− mice showed a very different gene expression profile (Fig. 8e). These data thus show that deficiency of CREB3L3 has no effect on hepatic gene expression in the absence of PPARα, indicating that the major liver phenotype triggered by CREB3L3 deficiency during ketogenic diet is dependent on PPARα.

Scatter plot analysis confirmed that the effects of PPARα and CREB3L3 deficiency on hepatic gene expression are very dissimilar, whereas the effect of PPARα deficiency and combined PPARα/CREB3L3 deficiency are similar (Additional file 1: Figure S2A). Venn diagram of significantly changed genes confirmed that deficiency of CREB3L3 leads to the up- and downregulation of a large set of genes that are not affected in the PPARα−/− or PPARα/CREB3L3−/− mice (Additional file 1: Figure S2B).

### Induction of mitogenic genes in CREB3L3−/− mice during ketogenic diet is mediated by PPARα

**Fig. 9:**
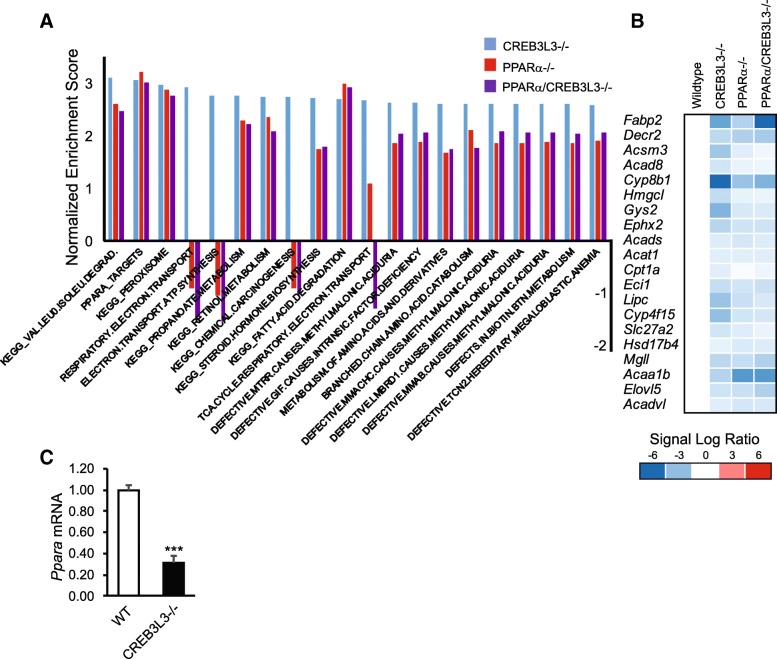
CREB3L3 deficiency during ketogenic diet leads to downregulation of PPARα targets. **a** Top 20 downregulated genesets in liver of CREB3L3−/− mice compared with wild-type mice, determined by geneset enrichment analysis and ranked according to normalized enrichment score (NES) (blue). The NES of the same genesets for the comparison between wild-type and PPARα−/− (red) or combined PPARα/CREB3L3−/− (purple) mice is shown as well. **b** Comparative gene expression analysis in liver of wild-type, PPARα−/−, CREB3L3−/−, and combined PPARα/CREB3L3−/− mice after a 4-day ketogenic diet. The genes shown are the 20 most highly enriched genes in CREB3L3−/− mice vs. wild-type mice that are part of the geneset PPARΑ targets. Red indicates upregulated, blue indicates downregulated. **c** Mean PPARα mRNA expression level in liver of wild-type and CREB3L3−/− mice after a 4-day ketogenic diet. Asterisk indicates significantly different from wild-type mice according to Student’s t-test (*P* < 0.001)

To obtain more insight into the functional pathways affected by CREB3L3 deficiency on ketogenic diet, we performed geneset enrichment analysis. Surprisingly, many of the most highly downregulated genesets represented pathways of fatty acid and/or amino acid metabolism, including peroxisome, PPARα targets, and fatty acid degradation (Fig. 9a). Enrichment scores for these latter genesets were similar in the PPARα−/− and PPARα/CREB3L3−/− mice (Fig. 9a). A heatmap of the geneset PPARα targets shows the consistent downregulation of PPARα target genes across the 3 groups of mice (Fig. 9b). These data suggest that CREB3L3 deficiency, as well as PPARα deficiency and combined PPARα/CREB3L3 deficiency, leads to reduced PPARα activity. In line with these data, the PPARα mRNA expression level was markedly reduced in the CREB3L3−/− mice (Fig. 9c).

**Fig. 10:**
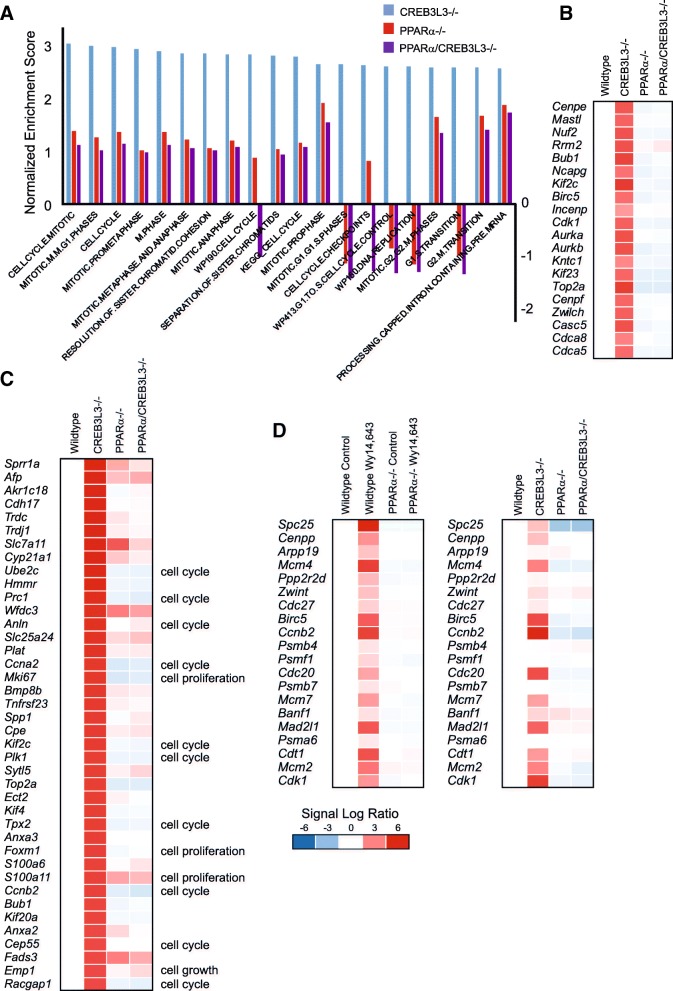
CREB3L3 deficiency during ketogenic diet leads to upregulation of the cell cycle. **a** Top 20 upregulated genesets in liver of CREB3L3−/− mice compared with wild-type mice, determined by geneset enrichment analysis and ranked according to normalized enrichment score (NES) (blue). The NES of the same genesets for the comparison between wild-type and PPARα−/− (red) or combined PPARα/CREB3L3−/− (purple) mice is shown as well. **b** Comparative gene expression analysis in liver of wild-type, PPARα−/−, CREB3L3−/−, and combined PPARα/CREB3L3−/− mice after a 4-day ketogenic diet. The genes shown are the 20 most highly enriched genes in CREB3L3−/− mice vs. wild-type mice that are part of the geneset CELL.CYCLE.MITOTIC. **c** Comparative gene expression analysis in liver of wild-type, PPARα−/−, CREB3L3−/−, and combined PPARα/CREB3L3−/− mice after a 4-day ketogenic diet, showing the top 40 most highly upregulated genes in CREB3L3−/− mice. **d** Comparative gene expression analysis in liver of wild-type mice, wild-type mice treated with Wy-14,643 for 5 days, PPARα−/− mice, and PPARα−/− treated with Wy-14,643 for 5 days (left panel), and wild-type, PPARα−/−, CREB3L3−/−, and combined PPARα/CREB3L3−/− mice after a 4-day ketogenic diet (right panel). The genes shown are the 20 most highly enriched genes upon Wy-14.643 treatment that are part of the geneset MITOTIC.M.M.G1 PHASE. Red indicates upregulated, blue indicates downregulated

Geneset enrichment analysis also underscored the dramatic effect of CREB3L3 deficiency on hepatic gene expression. Indeed, 538 genesets met the statistical significance cut-off of FDR q-value< 0.05, covering numerous biological processes, including immunity, cellular stress pathways, and DNA/RNA-related processes (not shown). Intriguingly, the 20 most upregulated genesets were all related to cell cycle/mitosis (Fig. 10a). Enrichment scores for these genesets were much lower in the PPARα−/− and PPARα/CREB3L3−/− mice, indicating the selective induction of cell cycle/mitosis-related genes in the CREB3L3−/− mice (Fig. 10a). A heatmap of the most enriched genes within the geneset Cell.Cycle.Mitotic demonstrates the pronounced upregulation of cell cycle genes in the CREB3L3−/− mice (Fig. 10b). Strikingly, the upregulation is completely abolished upon additional deficiency of PPARα, suggesting that PPARα mediates the induction of cell cycle genes in CREB3L3−/− mice on ketogenic diet (Fig. 10b). Consistent with the upregulation of cell cycle upon CREB3L3 deficiency, many of the most highly induced genes in the CREB3L3−/− mice on ketogenic diet were related to cell cycle (Fig. 10c). Again, the upregulation of these genes was almost completely abolished in the PPARα−/− mice.

In line with the known mitogenic effect of PPARα activation on hepatocyte proliferation, pharmacological activation of PPARα in vivo has been shown to cause the induction of numerous genes and proteins involved in cell cycle control [28], which is specifically mediated by mouse PPARα and not human PPARα [29]. Previously, we found that treating mice with the specific PPARα agonist Wy-14,643 markedly induced numerous genesets related to cell cycle [2]. A heatmap of the most highly enriched genes in the geneset Mitotic.M.M.G1 phase underscores the marked induction of cell cycle-related genes by Wy-14,643, which is entirely PPARα dependent (Fig. 10d). Strikingly, most of these genes are also highly upregulated in the CREB3L3−/− mice on ketogenic diet, which again is entirely PPARα dependent (Fig. 10d), indicating that the pronounced upregulation of the cell cycle in CREB3L3−/− mice is mediated by PPARα. Taken together, these data indicate that CREB3L3 deficiency uncouples the hepatoproliferative and lipid metabolic effects of PPARα.

## Discussion

In this paper we studied the effect of individual and combined PPARα and CREB3L3 deficiency on hepatic gene expression after a 16-h fast and a 4-day ketogenic diet. Under conditions of overnight fasting, the effect of PPARα deficiency and CREB3L3 deficiency on hepatic gene expression are largely independent, and only show a very limited degree of synergism. A small number of genes is under dual control of PPARα and CREB3L3, including *Fgf21* and *Mfsd2a*. Our data do not support a strong co-dependence of PPARα and CREB3L3 in hepatic gene regulation during fasting. By contrast, a strong interaction between PPARα and CREB3L3 exists during ketogenic diet feeding. Previously, it was shown that CREB3L3−/− mice on a ketogenic diet exhibit a strong phenotype characterized by hepatomegaly and steatohepatitis, and elevated expression of inflammatory marker genes [19, 25]. Here, using whole genome expression profiling, we corroborate these findings. In addition, we show that deficiency of CREB3L3 has virtually no effect on hepatic gene expression in the absence of PPARα, indicating that the major liver phenotype triggered by CREB3L3 deficiency during ketogenic diet is dependent on PPARα. Furthermore, we find that CREB3L3 has a dual impact on PPARα signalling during ketogenic diet. On the one hand, CREB3L3 deficiency leads to reduced expression of PPARα and PPARα target genes involved in fatty acid oxidation and ketogenesis. On the other hand, CREB3L3 deficiency leads to the marked activation of the hepatoproliferative effect of PPARα. Overall, our data suggest that CREB3L3 deficiency during ketogenic diet uncouples the mitogenic and lipid metabolic effects of PPARα in the liver.

It is unclear how CREB3L3 deficiency promotes liver damage and hepatoproliferation during ketogenic diet and how this effect is dependent on PPARα. It could be envisioned that deficiency of CREB3L3 disrupts a certain metabolic pathway, such as fatty acid oxidation or fatty acid elongation and desaturation, leading to accumulation of intermediate lipid species that ligand-activate PPARα and specifically stimulate the mitogenic action of PPARα. In addition, these lipid species may promote liver damage. Additionally, it is possible that CREB3L3 deficiency alters a specific metabolic pathway, possibly involving accumulation of damaging intermediates, and that these effects are dependent on an enzyme/factor whose expression is maintained by PPARα. Insofar as CREB3L3 and PPARα regulate the expression of many genes, it is not possible to pinpoint the exact causal gene(s) downstream of CREB3L3 and PPARα.

Other examples exist of the uncoupling of the mitogenic and metabolic actions of PPARα. For example, human PPARα upregulates genes involved in fatty acid oxidation but not the cell cycle, as shown by studies in mice carrying human PPARα [29, 30]. Another example is the activation of mouse PPARα by dietary n-3 poly-unsaturated fatty acids, which leads to upregulation of PPARα targets involved in lipid metabolism but does not trigger hepatocyte proliferation [31]. These findings strongly indicate that the mechanisms by which PPARα affects lipid metabolism and hepatocyte proliferation are distinct [29, 30]. Mechanistically, how ligand-activated PPARα could selectively activate mitogenic and not metabolic pathways is unclear but could be related to the SPPARM concept [32–34]. According to this concept, different PPAR agonists have only partially overlapping effects on gene expression based on selective receptor-coregulator interactions. Borrowing from this notion, it can be hypothesized that the epigenetic mechanisms that drive the PPARα-dependent activation of genes involved in fatty acid oxidation and ketogenesis are different from the epigenetic mechanisms that support the induction of mitogenic pathways by PPARα, and additionally that these mechanisms are differentially affected by CREB3L3 deficiency.

**Fig. 11:**
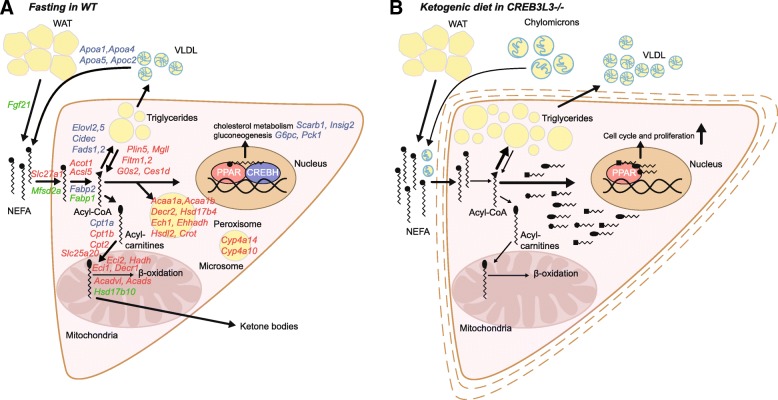
PPARα and CREB3L3 cooperate to regulate hepatic lipid metabolism. **a** The cartoon illustrates the distinct roles of CREB3L3 and PPARα in the regulation of hepatic lipid metabolism during fasting. CREB3L3 stimulates genes involved in lipoprotein metabolism (Apoc2, Apoa5, Apoa1), lipid storage (*Cidec*), fatty acid binding (*Fabp2*), fatty acid desaturation and elongation (*Fads1, Fads2, Elovl2, Elovl5*), gluconeogenesis (*Pck1,G6pc*), and fatty acid oxidation (*Cpt1a, Hsd17b10*). PPARα stimulates genes involved in peroxisomal fatty acid oxidation (*Acaa1, Decre2, Ehhadh, Ech1*), mitochondrial fatty acid oxidation (*Slc25a20, Cpt2, Acadvl, Hadh*), microsomal fatty acid oxidation (*Cyp4a10, Cyp4a14*), fatty acid binding and (de)activation (*Fabp1, Acsl5, Acot1*), triglyceride hydrolysis (*Plin5, Fitm1, G0 s2, Mgll*). Genes significantly decreased in CREB3L3−/− mice in the fasted state are in blue (IBMT *P*-value< 0.001). Genes significantly decreased in PPARα−/− mice in the fasted state are in red. Genes significantly decreased in both genotypes are shown in green. **b** Overview of the effect of CREB3L3 deficiency after 4 days of ketogenic diet feeding. It is hypothesized that CREB3L3 deficiency during ketogenic diet leads to accumulation of certain lipid species that (ligand) activate PPARα. In turn, PPARα activation leads to hepatocyte proliferation and hepatomegaly. Additional effects of CREB3L3 deficiency include steatosis and enhanced liver damage

Our data indicate that the roles of PPARα and CREB3L3 in the fasted state are very distinct, showing minimal overlap in target gene regulation (Fig. 11a). As shown by previous whole genome expression analyses and supported by the present paper, PPARα governs the expression of a large number of genes involved in fatty acid oxidation and ketogenesis, as well as other pathways of intracellular and extracellular lipid metabolism [2]. Reduced fatty acid oxidation and ketogenesis causes the commonly observed fasting-induced hypoketonemia and elevated plasma free fatty acid levels in PPARα−/− mice [9–11, 35]. In the liver, the non-oxidized fatty acids are diverted towards re-esterification, explaining the fasting-induced steatosis in PPARα−/− mice [9–11, 35]. By contrast, CREB3L3 targets apolipoproteins, including *Apoa4*, *Apoc2*, *Apoa5*, and *Apoa1* [17, 36]. Reduced expression of the lipoprotein lipase activators *Apoc2* and *Apoa5*, and of *Fgf21*, which at pharmacological doses has been shown to stimulate plasma triglyceride clearance [37], likely explains the elevated plasma triglyceride levels in CREB3L3−/− mice via reduced plasma triglyceride clearance [17, 25, 36, 38]. This is in line with our previous observation that CREB3L3 deficiency does not significantly influence triglyceride secretion [19]. Besides targeting lipoprotein metabolism, CREB3L3 regulates a relatively small number of genes involved in several distinct metabolic pathways, including *Fabp2* (fatty acid binding), *Cidec* (lipid storage), fatty acid desaturation (*Fads1*, *Fads2*), fatty acid elongation (*Elovl2*, *Elovl5*), and gluconeogenesis (*G6pc, Pck1*) (Fig. 11a) [15–18]. Our data do not support the notion that CREB3L3 has an important role during fasting in regulating genes involved in fatty acid oxidation, with the exception of *Cpt1a* and *Hsd17b10*. This is supported by our previous data, showing a lack of effect of CREB3L3 deficiency on ex vivo fatty acid oxidation and fatty acid oxidation genes [19]. In contrast, Nakagawa and colleagues observed that CREB3L3 deficiency in the fasted state reduced expression of many genes involved in fatty acid oxidation, showing synergy with PPARα [25]. The reason for this discrepancy is not clear, but could be related to the different duration of fasting (16 h vs. 24 h). Interestingly, *Cpt1a* expression was not downregulated in the fasted state in PPARα−/− mice, which we had also observed in another set of samples [12], despite it being considered as the prototypical PPARα target gene. It can be hypothesized that the stimulatory effect of PPARα agonists on *Cpt1a* expression may be partly mediated by induction of CREB3L3.

An intriguing question is why CREB3L3 deficiency leads to a pronounced phenotype in mice fed a ketogenic but has much more limited effects in fasted mice. Direct comparison of hepatic gene expression in wild-type mice after fasting and ketogenic diet showed that expression of SREBP1 and its target genes involved in lipogenesis and cholesterogenesis was much higher after the ketogenic diet than after fasting (not shown). Previously, it was shown that CREB3L3 is a negative regulator of SREBP-1c production and hepatic lipogenesis [39], which is in line with our observation that genes involved in lipogenesis/cholesterogenesis are highly elevated in CREB3L3−/− mice under regular fasting conditions. Hence, placing CREB3L3−/− mice on a ketogenic diet is expected to lead to markedly increased lipogenesis/cholesterogenesis, which in turn may lead to the generation of a specific (set of) lipids that could trigger the mitogenic effect of PPARα (Fig. 11b). The upregulation of cholesterogenesis upon CREB3L3 deficiency in the fasted state is seemingly at odds with a previous study that suggested that CREB3L3 stimulates lipogenesis and cholesterogenesis [40]. However, closer inspection at the individual gene levels shows substantial correspondence and indicates that CREB3L3 downregulates SREBP-dependent genes.

Despite lower expression of *Cidec*, which promotes lipid droplet formation [41, 42], PPARα−/−, CREB3L3−/−, and PPARα/CREB3L3−/− mice have elevated hepatic triglyceride levels. Similarly, expression of *Plin5*, which also promotes hepatic fat storage [43], is lower in PPARα−/− mice, despite these mice showing more pronounced steatosis. Accordingly, these data suggest that the elevated hepatic triglycerides in the PPARα−/−, CREB3L3−/−, and PPARα/CREB3L3−/− mice are not mediated by changes in *Cidec* and *Plin5* expression. It should be noted that an increase in liver triglycerides does not necessarily have to be accompanied by elevated hepatic expression of *Cidec* and/or *Plin5*.

One limitation of our study is that we used whole body PPARα−/− and CREB3L3−/− mice. Ideally, it would have been better to use liver-specific PPARα and CREB3L3 deficient mice. Nevertheless, due to the high expression of PPARα and CREB3L3 in liver, we believe the results presented here reflect the hepatic function of the two transcription factors [2, 13]. An additional limitation is that we did not unveil the molecular details of the interaction between PPARα and CREB3L3 during ketogenic diet. These aspects should be further addressed in future studies.

## Conclusion

We find that PPARα and CREB3L3 regulate distinct genes in the liver during fasting, with the exception of a limited number of common targets such as *Fgf21*. Strikingly, deficiency of CREB3L3 in mice during ketogenic diet uncouples the hepatoproliferative and metabolic effects of PPARα. Our data underscore the distinct functions of PPARα and CREB3L3 in the regulation of hepatic gene expression.

## Methods

### Animal experiments

CREB3L3−/− mice were backcrossed onto a C57BL/6 background at least 10 times [17]. PPARα−/− mice that had been backcrossed on a pure C57Bl/6J background for more than 10 generations were acquired from Jackson Laboratories (no. 008154, B6;129S4-Pparatm1Gonz/J) [44]. The two lines were interbred to generate combined PPARα/CREB3L3−/− mice. Mice were housed in a specific pathogen free facility at the Weill Cornell Medical College on a 12 h light/dark cycles and fed ad libitum standard chow diet (PicoLab Rodent diet 20, #5058, Lab diet). The four different mouse lines (wild-type, PPARα−/−, CREB3L3−/−, and PPARα/CREB3L3−/−) were either fasted for 16 h or fed a ketogenic diet for 4 days (# F3666, Bio-Serv). The mice used for experiments were all male and approximately 8 weeks old. The euthanasia was carried out at around 10 a.m., with the ketogenic diet group being non-fasted (ad libitum fed). Blood was taken by orbital puncture under isoflurane anesthesia, followed by euthanasia of the mice by cervical dislocation. Tissues were excised and immediately frozen in liquid nitrogen followed by storage at − 80 °C.

For the adenoviral-mediated CREB3L3 overexpression, two-month-old male mice were injected intravenously via the tail vein at a dose of 3 × 10^9 particles of the adenoviruses per g body weight in 0.15 ml of saline. Mice injected with GFP-expressing adenovirus were used as control. Mice were euthanized four days after adenovirus injection and livers of three mice per group were used for whole genome expression profiling as detailed below. All animal experiments were approved by the Institutional Animal Care and Use Committee at Weill Cornell Medical College (Protocol #2012–0048) and performed in accordance with the approved guidelines.

### Biochemical assays

Plasma triglycerides, non-esterified fatty acids, ketone bodies, alanine aminotransferase, and FGF21 concentrations were determined using assay kits (Serum Triglyceride Determination Kit, Sigma; NEFA-HR (2), Wako Chemicals; Autokit Total Ketone Bodies, Wako Chemicals; ALT Kit, Bio-Quant; Mouse/Rat FGF-21 Quantikine ELISA Kit, R&D Systems; Human FGF-21 Quantikine ELISA Kit, R&D Systems). Lipids were extracted from liver tissues with chloroform/methanol mixture (2:1 *v*/v), as described previously [45].

### Transcriptomics

Microarray analysis was performed on liver samples using 3–4 biological replicates per group. Total RNA was extracted from cells using TRIzol reagent (Life Technologies, Bleiswijk, The Netherlands) and subsequently purified using the RNeasy Micro kit (Qiagen, Venlo, The Netherlands). RNA integrity was verified with RNA 6000 Nano chips on an Agilent 2100 bioanalyzer (Agilent Technologies, Amsterdam, The Netherlands). Purified RNA (100 ng) was labelled with the Ambion WT expression kit (Carlsbad, CA) and hybridized to an Affymetrix Mouse Gene 1.1 ST array plate (Affymetrix, Santa Clara, CA). Hybridization, washing, and scanning were carried out on an Affymetrix GeneTitan platform according to the manufacturer’s instructions. Normalized expression estimates were obtained from the raw intensity values applying the robust multi-array analysis preprocessing algorithm available in the Bioconductor library AffyPLM with default settings [46, 47]. Probe sets were defined according to Dai et al. [48]. In this method probes are assigned to Entrez IDs as a unique gene identifier. In this study, probes were reorganized based on the Entrez Gene database, build 37, version 1 (remapped CDF v22). The *P* values were calculated using an Intensity-Based Moderated T-statistic (IBMT) [49]. Genes were defined as significantly changed when *P* < 0.001.

Geneset enrichment analysis (GSEA) was used to identify gene sets that were enriched among the upregulated or downregulated genes [50]. Genes were ranked based on the IBMT-statistic and subsequently analyzed for over- or underrepresentation in predefined genesets derived from Gene Ontology, KEGG, National Cancer Institute, PFAM, Biocarta, Reactome and WikiPathways pathway databases. Only genesets consisting of more than 15 and fewer than 500 genes were taken into account. Statistical significance of GSEA results was determined using 1000 permutations.

### Statistical analysis

Statistical analysis of the transcriptomics data was performed as described in the previous paragraph. Statistical analysis of the other parameters was performed by two-way ANOVA and Student’s t-test. Data are presented as mean ± SEM. *P* < 0.05 was considered statistically significant.

## Additional file

Additional file 1: **Table S1.** List of 34 genes that were commonly downregulated in livers of PPARα−/−, CREB3L3−/−, and PPARα/CREB3L3−/− mice in the fasted state. **Table S2.** Specific functions of the genes connected to cell cycle illustrated in Figs. 10b and c. **Figure S1.** PPARα and CREB3L3 regulate distinct pathways in liver during fasting. **Figure S2.** Similar effects of PPARα and combined PPARα/CREB3L3 ablation on hepatic gene expression. (PDF 1161 kb)

## Abbreviations

CREB3L3: cAMP-Responsive Element Binding Protein 3-Like 3
GSEA: Gene Set Enrichment Analysis
NEFA: non-esterified fatty acids
PPAR: Peroxisome Proliferator-Activated Receptor
SPPARM: Selective PPAR Modulator
