# Supplementary material for: Transcriptional profiling of PPARα−/− and CREB3L3−/− livers reveals disparate regulation of hepatoproliferative and metabolic functions of PPARα
Source: BMC Genomics. 2019 Mar 11;20:199. doi: 10.1186/s12864-019-5563-y (PMC6416987; doi:10.1186/s12864-019-5563-y)
Supplement: Supplementary file 1 — Table S1. List of 34 genes that were commonly downregulated in livers of PPARα−/−, CREB3L3−/−, and PPARα/CREB3L3−/− mice in the fasted state. Table S2. Specific functions of the genes connected to cell cycle illustrated in Figs. 10b and c. Figure S1. PPARα and CREB3L3 regulate distinct pathways in liver during fasting. Figure S2. Similar effects of PPARα and combined PPARα/CREB3L3 ablation on hepatic gene expression. (PDF 1161 kb) [file 12864_2019_5563_MOESM1_ESM.pdf]

Supplementary Material

Supplemental Table 1. List of 34 genes that were commonly downregulated in livers of PPARα-/-, CREB3L3-/-, and PPARα/CREB3L3-/- mice in the fasted state (IBMT P-value<0.001).

Mfsd2a	Major Facilitator Superfamily Domain Containing 2A
Mtnr1a	Melatonin Receptor 1A
Fgf21	Fibroblast Growth Factor 21
Lamb3	Laminin Subunit Beta 3
Sema5b	Semaphorin 5B
Xrcc3	X-Ray Repair Cross Complementing 3
D630045J12Rik	RIKEN cDNA D630045J12 gene
Crip2	Cysteine Rich Protein 2
Tlr12	Toll-like Receptor 12
Srebf1	Sterol Regulatory Element Binding Transcription Factor 1
Kcnk1	Potassium Two Pore Domain Channel Subfamily K Member 1
Tmem28	Transmembrane Protein 28
Rtfdc1	Replication Termination Factor 2
Dpy19l3	Dpy-19 Like C-Mannosyltransferase 3
Hsd17b10	Hydroxysteroid 17-Beta Dehydrogenase 10
Gm10851	Predicted gene 10851
Sntg2	Syntrophin Gamma 2
Tram2	Translocation Associated Membrane Protein 2
Tmem134	Transmembrane Protein 134
Suclg1	Succinate-CoA Ligase Alpha Subunit
Sulf2	Sulfatase 2
Fam73b	Mitoguardin 2
Nat1	N-acetyl transferase 1
Tmem184a	Transmembrane protein 184a
Sun2	Sad1 and UNC84 domain containing 2
Sel1l3	Sel-1 suppressor of lin-12-like 3 (C. elegans)
Cog4	Component Of Oligomeric Golgi Complex 4
Rtn4ip1	Reticulon 4 Interacting Protein 1
Rmdn3	Regulator Of Microtubule Dynamics 3
Nsmf	NMDA Receptor Synaptonuclear Signaling And Neuronal Migration Factor
Ldha	Lactate dehydrogenase A
Ak2	Adenylate kinase 2
St3gal3	*S*T3 beta-galactoside alpha-2,3-sialyltransferase 3
Oard1	O-acyl-ADP-ribose deacylase 1

Supplemental Table 2. Specific functions of the genes connected to cell cycle illustrated in figures 10B and C.

Cenpe, Centrosome-associated protein E	required for stable spindle microtubule capture at kinetochores	Promoting cell cycle progression
Mastl, microtubule-associated serine/threonine kinase		Possibly Pro
Nuf2, NDC80 kinetochore complex component NUF2	Yeast Nuf2 disappears from the centromere during meiotic prophase when centromeres lose their connection to the spindle pole body, and plays a regulatory role in chromosome segregation	Likely pro
Rrm2	catalyzes the formation of deoxyribonucleotides from ribonucleotides	Pro
Bub1, BUB1 mitotic checkpoint serine/threonine kinase	The encoded protein functions in part by phosphorylating members of the mitotic checkpoint complex and activating the spindle checkpoint	Pro
Ncapg, non-SMC condensin I complex subunit G	subunit of condensin complex, which is involved in condensation and stabilization of chromosomes during mitosis	Likely pro
Kif2c, Kinesin family member 2c	depolymerize microtubules at the plus end, thereby promoting mitotic chromosome segregation	Pro
Birc5, baculoviral IAP repeat containing 5	inhibitor of apoptosis (IAP) gene family, which encode negative regulatory proteins that prevent apoptotic cell death	Possibly Pro
Incep, inner centromere protein		Pro
Cdk1		Pro
Aurka, aurora kinase A	involved in microtubule formation and/or stabilization at the spindle pole during chromosome segregation	Pro
Aurkb, same as Aurka		Pro
Kntc1, kinetochore associated 1	involved in mechanisms to ensure proper chromosome segregation	Pro
Kif23, kinesin family member 23	This protein has been shown to cross-bridge antiparallel microtubules and drive microtubule movement in vitro	Pro
Top2a, DNA topoisomerase II alpha		Pro
Cenpf, centromere protein F		Pro
Zwilch, zwilch kinetochore protein		Likely Pro
Casc5, kinetochore scaffold	interacts with at least 5 different kinetochore proteins and two checkpoint kinases	Likely Pro
Cdca8, cell division cycle associated 8	Spindle formation	Pro
Cdca5 – same as Cdca8		Pro
Ube2c, ubiquitin conjugating enzyme E2 C	The encoded protein is required for the destruction of mitotic cyclins and for cell cycle progression	Pro
Prc1, protein regulator of cytokinesis 1	present at high levels during the S and G2/M phases of mitosis but its levels drop dramatically when the cell exits mitosis and enters the G1 phase. It is located in the nucleus during interphase, becomes associated with mitotic spindles in a highly dynamic manner during mitosis, and localizes to the cell mid-body during cytokinesis. This protein has been shown to be a substrate of several cyclin-dependent kinases (CDKs). It is necessary for polarizing parallel microtubules and concentrating the factors responsible for contractile ring assembly	Pro
Anln, anillin actin binding protein	This gene encodes an actin-binding protein that plays a role in cell growth and migration, and in cytokinesis	Likely Pro

Ccna2, cyclin A2	his protein binds and activates cyclin-dependent kinase 2 and thus promotes transition through G1/S and G2/M	Pro
Mki67, marker of proliferation Ki-67	"necessary for cellular proliferation"	Possibly Pro
Plk1, polo like kinase 1	Depletion of this protein in cancer cells dramatically inhibited cell proliferation and induced apoptosis	Pro
Tpx2, microtubule nucleation factor	controls spindle integrity, genome stability	Likely Pro
Foxm1, forkhead box M1	The encoded protein is phosphorylated in M phase and regulates the expression of several cell cycle genes, such as cyclin B1 and cyclin D1	Pro
S100a11, S100 calcium binding protein A11	involved in the regulation of a number of cellular processes such as cell cycle progression and differentiation	Possibly Pro
Ccnb2, cyclin B2	essential components of the cell cycle regulatory machinery	Likely pro
Cep55, centrosomal protein 55		Possibly Pro
Emp1, epithelial membrane protein 1		?
Racgap1, Rac GTPase activating protein	This protein plays a regulatory role in cytokinesis, cell growth, and differentiation	Possibly Pro

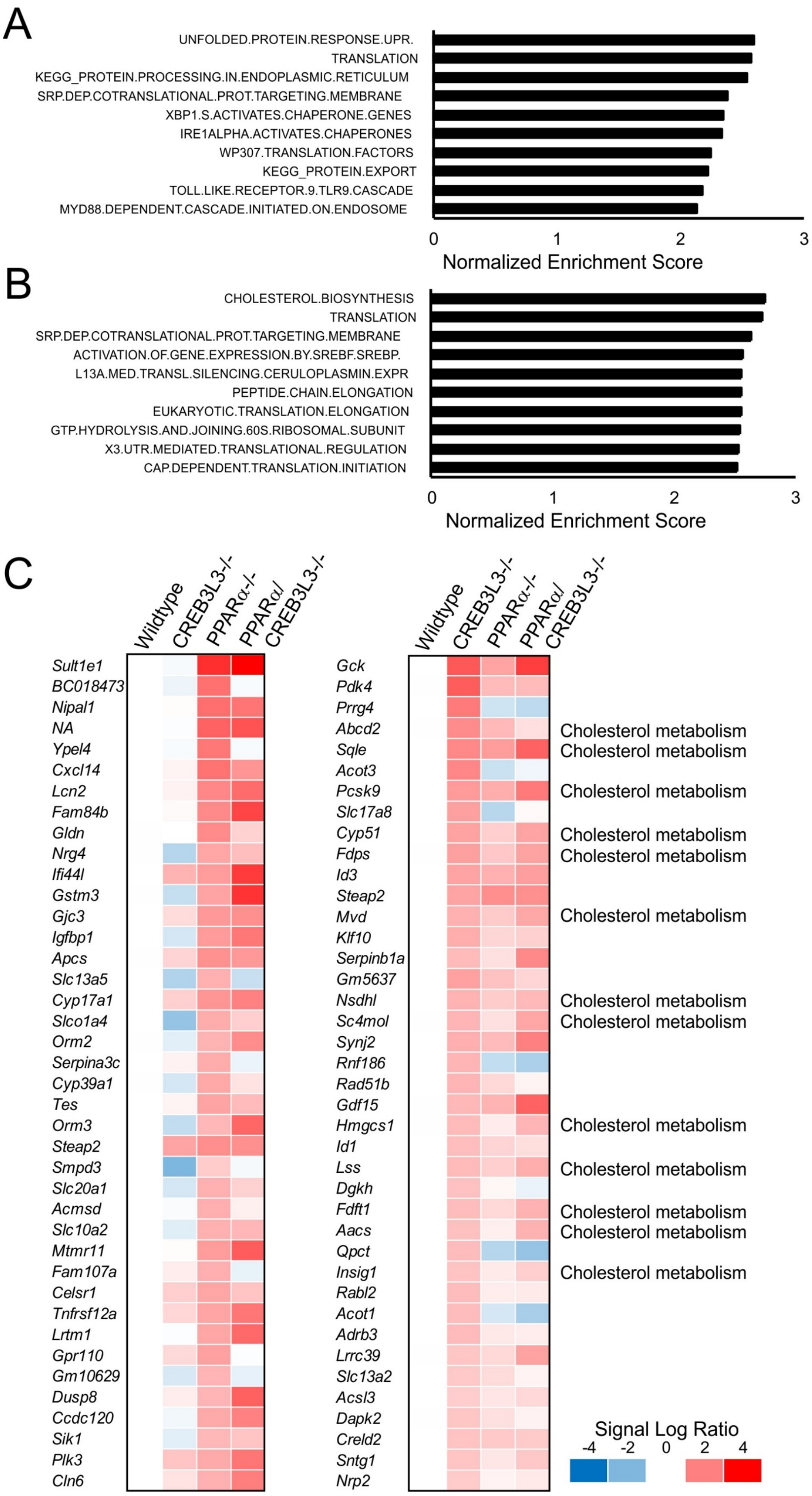

Supplemental figure 1. PPARα and CREB3L3 regulate distinct pathways in liver during fasting. Top 10 upregulated gene sets in liver of (A) PPARα-/- mice and (B) CREB3L3-/- mice compared to wildtype mice, as determined by gene set enrichment analysis. Gene sets were ranked according to normalized enrichment score. C) Comparative gene expression analysis in liver of wildtype, PPARα-/-, CREB3L3-/-, and PPARα/CREB3L3-/- mice after a 16h fast, showing the top 40 most highly upregulated genes in PPARα-/- mice (left panel) and CREB3L3-/- mice (right panel).

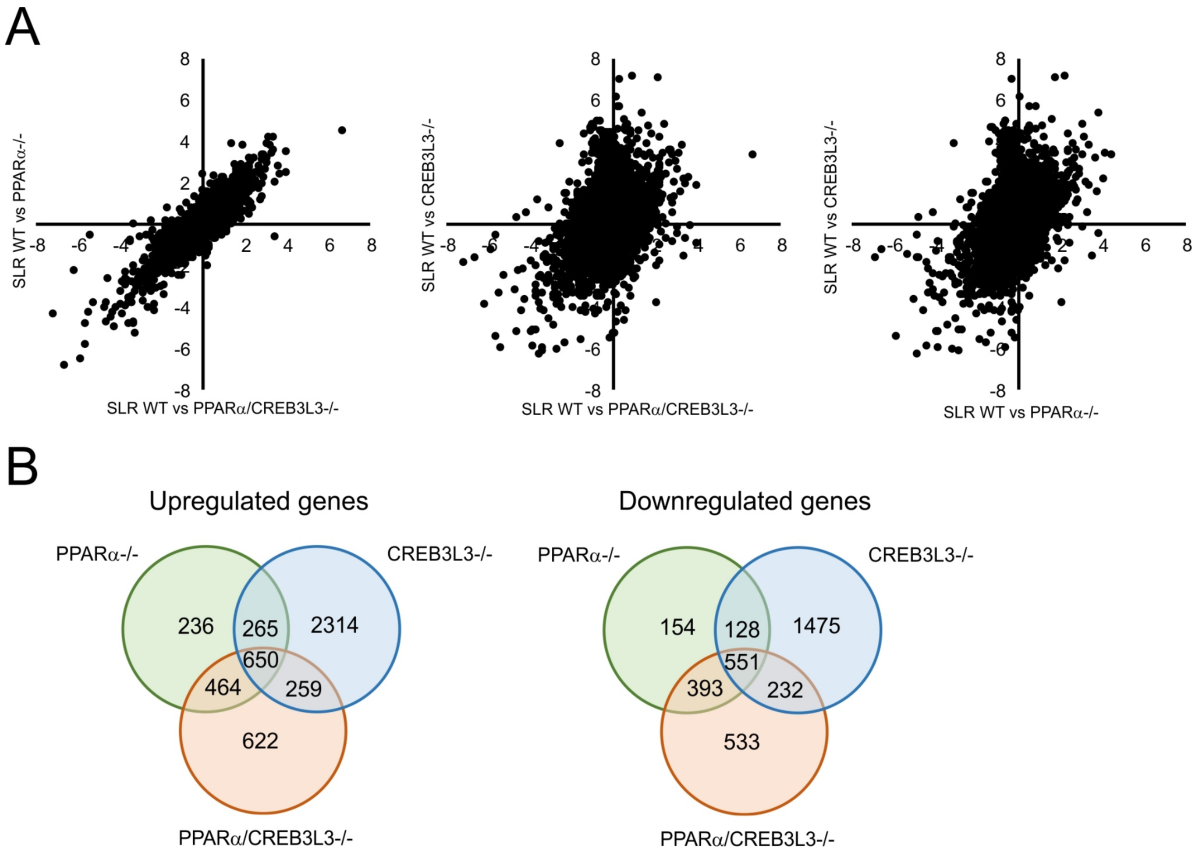

Supplemental figure 2. Similar effects of PPARα and combined PPARα/CREB3L3 ablation on hepatic gene expression. A) Correlation plot showing comparative hepatic gene expression changes in PPARα-/-, CREB3L3-/-, and combined PPARα/CREB3L3-/- mice in relation to wildtype mice after a 4 day ketogenic diet (expressed as signal log ratio, SLR). B) Venn diagram showing overlap in upregulated genes (left panel) and downregulated genes (right panel) in PPARα-/-, CREB3L3- /-, and combined PPARα/CREB3L3-/- mice, in comparison with wildtype mice (IBMT P value<0.001).
